# Medical Opinion and Movement

**Published:** 1906-11-24

**Authors:** 


### Medical Opinion and Movement.

Mr. Henry Sewill has made an excellent suggestion in regard to the sales of patent medicine scandal which is doing so much injury to the health of the people in these islands. He suggests the appointment of a royal commission in order to bring out, by evidence, the infamy of quackery and the dangers of the quack medicine trade. He holds the view, that, the evidence which could be produced before such a commission would enable legislation to be enacted, whereby the greater part of the trade in quack medicines could be brought within the scope of the ordinary criminal law. He points out with force and truth, that, if a royal commission on patent medicines is appointed, the exposure of the facts must bring shame to some of the wealthier newspaper proprietors, who take so large a share of the enormous sums spent upon advertisements of quack remedies, although they must be fully aware of the evils, which the sale of these commodities inflicts upon great masses of the people. We cordially support the demand for a royal commission, and hope that the present government may decide to appoint one without delay.

The knighthood conferred by the King upon Sir John Tweedy, the eminent oculist, has given the greatest satisfaction to the whole medical profession. Sir John Tweedy was one of the most popular and able presidents of the Royal College of Surgeons; he has given many years of his life to the service of the poor in connection with several of the hospitals; more than thirty years ago he was a power in medical journalism as a member of the staff of the *Lancet*; as an administrator he has few equals in the medical profession; and his pleasant personality and sound business sense make him invaluable as a colleague upon committees, especially where the work is difficult and requires the exercise of sound judgment and great tact. We hope that Sir John Tweedy may long be spared to enjoy the honour which he has well earned, and to take a leading part in the councils of the medical profession of which he is so popular and representative a leader. Our congratulations are due too to Sir Alfred Fripp, K.C.V.O., to whom Guy's Hospital owes much, and who has shown great ability as a surgeon and won considerable reputation by his conduct of many difficult cases.

An important meeting of the Marylebone Division of the British Medical Association will be held at the rooms of the Medico-Chirurgical Society, 20 Hanover Square, W, on Monday, December 3, at 8.30 p.m. The Marylebone Division has very properly been impressed with the importance of raising, during the winter, discussions on economical problems in the practice of medicine. The discussion on the 3rd prox. will centre round a resolution, as follows: that it is desirable that concerted action should be taken to lessen the large amount of gratuitous medical attendance granted to many sections of the community; that this eleemosynary service tends to diminish the thrift and self-respect of the public, lessens the esteem in which medical science is held, and has a detrimental effect upon the position of many members of the profession. We are of course entirely in sympathy with the views embodied in these resolutions. It would be well, however, in our judgment, to add a rider to the resolution, to the effect, that, the supporters of the voluntary hospitals are becoming fully alive to the evils attending the continuous increase in the free medical treatment granted by these institutions. For financial reasons, these authorities must concert measures, in conjunction with their visiting staffs, promptly to reduce the number of patients, and to confine this free treatment to those cases, only, whose social circumstances entitle them to receive it.

The practice of keeping incurable cases at Netley Hospital, which contains 900 beds, less than half of which are occupied in ordinary times, has much to be said in its favour. Lord Robert Cecil did good service by championing the cause of those soldiers who, in the course of their service, through illness or disease, became unable to work before they have served with the colours long enough to entitle them to pension. The total cost of maintaining these Netley cases was admitted to be only £3,000 a year, and it was further admitted that if these invalid soldiers are not retained at Netley, there is no place open to many of them except the workhouse. A radical government is in power; the history of all armies shows that the mortality and wastage from disease is far greater than from injuries and wounds; and yet the English nation under Mr. Haldane's leadership, by turning these chronic cases out of Netley Hospital into the workhouse, is held up to the just scorn of other nations by being made to appear so cruelly unjust in its treatment of its soldiers. What can be said in defence of an employer like the British Government, which conducts the most dangerous of all businesses, that of war, whilst it imposes heavy liabilities upon British employers of labour in regard to accidents to their employees, but refuses to make a reasonable provision for the care and sustenance of the British soldier who, from no fault of his own, is struck down by disease, and permanently disabled from earning his own living? This is no question of party politics, but it is one which affects the honour of every man and woman in these islands. It is said that the present House of Commons is largely socialistic in its sympathies. This must surely be wrong, when the British House of Commons, by 259 votes to 61, turns the British soldier out of Netley Hospital and consigns him to the workhouse in order to save a paltry £3,000 a year. What is the Labour party doing that the voices of its leaders in the House of Commons should remain silent, whilst this grave injustice is done to the soldier, who, after all, deserves the most tender consideration of his countrymen and countrywomen in the day of illness and misfortune?

